# *Dirofilaria immitis* in Dog Imported from Venezuela to Chile

**DOI:** 10.3201/eid2902.221427

**Published:** 2023-02

**Authors:** Cristian A. Alvarez Rojas, Beatriz Cancino-Faure, Pablo Lillo, María Luisa Fernández, Alejandro Piñeiro González, Alonso Flores Ramírez

**Affiliations:** Escuela de Medicina Veterinaria, Facultad de Agronomía e Ingeniería Forestal, Facultad de Ciencias Biológicas y Facultad de Medicina, Pontificia Universidad Católica de Chile, Santiago, Chile (C.A. Alvarez Rojas, P. Lillo);; Laboratorio de Microbiología y Parasitología, Departamento de Ciencias Preclínicas, Facultad de Medicina, Universidad Católica del Maule, Talca, Chile (B. Cancino-Faure, A. Piñeiro González);; Hospital Veterinario Mi Mascota, Santiago (M.L. Fernández);; Sociedad Chilena de Cardiología Veterinaria, Santiago (A. Flores Ramírez)

**Keywords:** Dirofilaria immitis, zoonoses, vector-borne infections, parasites, nematode, mosquitoes, canine dirofilariosis, dog, Venezuela, Chile

## Abstract

We report a case of *Dirofilaria immitis* nematode infection in a dog imported from Venezuela that had been living for 2 years in Santiago, Chile, where this parasite had not been reported before. Our findings warrant surveillance for all dogs imported to Chile, given that suitable conditions exist for establishing this parasite.

*Dirofilaria immitis*, a species of zoonotic parasitic nematode transmitted by mosquitoes, causes canine dirofilariosis. These nematodes are usually found in countries with temperate and tropical climates and are endemic throughout Europe and in the southeastern regions of Asia and Africa (*1*). In the Americas, *D. immitis* nematodes are present in all countries and territories except Chile and Uruguay (*2*). We report a case of a female dog born in Venezuela and imported to Santiago, Chile, where she lived for 2 years before having *D. immitis* infection diagnosed in January 2022. 

The dog was a 5.5-year-old Shar-pei who was brought to 2 veterinary clinics in Santiago during December 2021–January 2022. The initial cause of the consultation was vulvar discharge evolving to vomiting, melena, and general discomfort. Initially, the dog’s health improved after treatment with enrofloxacin (5 mg/kg). However, the animal’s condition deteriorated after 1 week (in January 2022). Ultrasound examination showed the presence of a fetus for which no heartbeat was detected. Blood work showed severe anemia, kidney failure, and the presence of microfilariae at the blood smear examination. An echocardiogram examination showed signs compatible with tricuspid valve insufficiency, but no nematodes were observed. A blood transfusion was performed before surgery to remove the uterus, which showed maceration of the fetus. The dog was euthanized days after surgery.

We used 1 mL of a blood sample collected with EDTA from the dog to perform the modified Knott’s test; we measured microfilariae under microscopic examination by using Leica Application Suite 3.4.0 software (Leica Microsystems, https://www.leica-microsystems.com). The average length of 10 microfilariae was 285.6 μm and average width was 6.5 μm (Table), which agrees with findings by Foreyt (*3*), who reported a length of 270–325 μm and a width of 6.7–7.0 μm for *D*. *immitis* microfilariae. However, data on the length and width of microfilariae reported in the literature vary considerably (*4*). Amplification and sequencing of DNA from canine microfilariae are required to correctly identify the species causing the infection (*5*,*6*). Therefore, we centrifuged an aliquot (500 μL) of the fresh blood sample with EDTA and isolated DNA from the sediment by using the E.Z.N.A. Tissue DNA Kit (Omega Bio-Tek, https://www.omegabiotek.com) according to the manufacturer’s instructions. We used the DNA as a template for PCR with GoTaq DNA Polymerase (Promega, https://www.promega.com) and amplified a section of the mitochondrial cytochrome oxidase I (COI) with the primers COIintF (TGATTGGTGGTTTTGGTAA) and COIintR (ATAAGTACGAGTATCAATATC) (*5*) and a section of the 12S rDNA with the primers 12SF (GTTCCAGAATAATCGGCTA) and 12SR (ATTGACGGATG(AG)TTTGTACC) (*6*). We sequenced PCR products in both directions with the same PCR primers in Macrogen Online Sequencing Order System (https://dna.macrogen.com). We used the acquired sequences as input in BLAST (http://blast.ncbi.nlm.nih.gov) for comparison. The sequence for the *cox1* (OP811228) showed 100% homology with *D*. *immitis* entries deposited in GenBank from Spain (accession no. LC107816), Slovenia (accession no. OP494255), Hungary (accession no. KM452920), Thailand (accession no. MW577348), and China (accession no. EU159111). Moreover, the sequence acquired from the 12S rDNA (OP819559) showed 100% homology with GenBank entries identified as *D*. *immitis* from French Guiana (accession nos. MT252014), Myanmar (accession nos. OL714336), Thailand (accession no. MW512514), Brazil (accession nos. MZ678855), France (accession nos. MZ435877), and China (accession no. EU182327). Therefore, both sequences confirmed the diagnosis of *D*. *immitis* infection.

**Table:** Length and width of 10 microfilariae fixed in formalin and measured using the modified Knott’s test, in a dog imported from Venezuela with *Dirofilaria immitis* infection, Chile, January 2022

Microfilariae	Length, μm	Width, μm
1	287.8	6.0
2	295.5	7.2
3	280.2	7.6
4	281.8	6.6
5	277.7	7.3
6	295.7	6.1
7	275.3	5.4
8	284.0	6.2
9	289.7	6.6
10	288.9	6.0
Average	285.6	6.5

The dog was brought into Chile from Venezuela 2 years before the incidental finding of microfilariae in an examination of blood prompted by complications caused by a uterine infection and kidney failure. This finding is relevant because, in recent years, human migration from Venezuela into Chile and other Latin America countries has risen, and the role of pets transported with humans as a reservoir of vector-borne pathogens has not been investigated. More than 6 million people have left Venezuela because of ongoing economic, political, and humanitarian crises (*7*); of those, >448,138 live in Chile (*8*). No record exists of how many dogs have entered Chile from Venezuela, and no requirement exists for testing for *D*. *immitis* infection in dogs arriving in Chile. Little information is available regarding the prevalence of *D. immitis* infection in Venezuela; an older report indicates a 4%–29% prevalence of *D. immitis* infection in hunting dogs in Aragua State based on analysis using the modified Knott´s test (*9*), and a second report indicates a 15.2% prevalence in Sucre State based on analysis using the modified Knott‘s test and antigen detection (*10*).

Unofficial reports from veterinarians in Santiago have mentioned detecting structures resembling adult specimens of nematodes in echocardiograms of some dogs brought into Chile from Venezuela. Autochthonous cases may occur soon in Chile, where mosquitoes capable of acting as vectors are present and suitable climatic conditions for developing the transmission cycle exist. Furthermore, the sizeable population of free-roaming dogs in Chile can act as a reservoir for the parasite. Since *D. immitis* nematodes are not endemic in Chile, little knowledge of the infection exists among veterinarians. Massive screening of dogs that arrive in Chile from *D*. *immitis*–endemic countries is urgently needed.
